# A Qualitative Transcriptional Signature for Predicting Recurrence Risk for High-Grade Serous Ovarian Cancer Patients Treated With Platinum-Taxane Adjuvant Chemotherapy

**DOI:** 10.3389/fonc.2019.01094

**Published:** 2019-10-18

**Authors:** Yixin Liu, Zheyang Zhang, Tianhao Li, Xin Li, Sainan Zhang, Ying Li, Wenyuan Zhao, Yunyan Gu, Zheng Guo, Lishuang Qi

**Affiliations:** ^1^Basic Medicine College, Harbin Medical University, Harbin, China; ^2^College of Bioinformatics Science and Technology, Harbin Medical University, Harbin, China; ^3^Key Laboratory of Ministry of Education for Gastrointestinal Cancer, Department of Bioinformatics, School of Basic Medical Sciences, Fujian Medical University, Fuzhou, China; ^4^Key Laboratory of Medical Bioinformatics, Fuzhou, China

**Keywords:** ovarian cancer, platinum chemotherapy, taxane chemotherapy, predictive signature, relative expression orderings

## Abstract

Resistance to platinum and taxane adjuvant chemotherapy (ACT) is the main cause of the recurrence and poor prognosis of high-grade serous ovarian cancer (HGS-OvCa) patients receiving platinum-taxane ACT after surgery. However, currently reported quantitative transcriptional signatures, which are commonly based on risk scores summarized from gene expression, are unsuitable for clinical application because of their high sensitivity to experimental batch effects and quality uncertainties of clinical samples. Using 226 samples of HGS-OvCa patients receiving platinum-taxane ACT in TCGA, we developed a qualitative transcriptional signature, consisting of four gene pairs whose within-samples relative expression orderings could robustly predict patient recurrence-free survival (RFS). In two independent test datasets, the predicted non-responders had significantly shorter RFS than the predicted responders (log-rank *p* < 0.05). In a test dataset containing data for patient pathological response state, the signature reclassified 12 out of 22 pathological complete response patients as non-responders and two out of 16 pathological non-complete response patients as responders. Notably, the 12 predicted non-responders in the pathological complete response group had significantly shorter RFS than the predicted responders (log-rank *p* = 0.0122). This qualitative transcriptional signature, which is insensitive to experimental batch effects and quality uncertainties of clinical samples, can individually identify HGS-OvCa patients who are more likely to benefit from platinum-taxane adjuvant chemotherapy.

## Introduction

Epithelial ovarian cancer has the highest mortality rate of all gynecologic cancers. The majority of patients with ovarian cancer are diagnosed as high grade (i.e., grade 2–3) (1). The standard treatment for high-grade serous ovarian cancer (HGS-OvCa) is surgery and platinum-based adjuvant chemotherapy (ACT) which is typically administered concurrently with taxane (2), denoted as platinum-taxane ACT. However, more than 70% of HGS-OvCa patients will develop recurrent disease within a few years after receiving platinum-taxane ACT, resulting in a 5-year survival rate of <40% (1, 3). Therefore, it is necessary to develop a predictive signature to distinguish responders who should receive platinum-taxane ACT from non-responders who should receive alternative therapies.

Recently, some studies have been devoted to developing predictive signatures for platinum-taxane ACT, based on gene expression profiles of primary tumor samples (4–6). However, most of the reported transcriptional signatures, such as 40-gene signature (5) and 23-gene signature (6), are based on risk scores to distinguish patients with shorter and longer survival rates after receiving platinum-based ACT, and defined them as non-responders and responders to platinum-based ACT, respectively. While, responders (or non-responders) predicted by these signatures may be resistant (or sensitive) to platinum-based ACT because of a low (or high) degree of malignancy of their own cancer cells (7), the signature is not directly applied to predict the sensitivity of platinum or taxane. Therefore, information about a patient's pathological response should be utilized to increase the relevance of signatures to platinum-taxane ACT.

More importantly, our recent work (8) has shown that this kind of signature, based on risk scores summarized from gene expression measurements of the signature genes, tends to be impractical in clinical settings because its application requires the pre-collection of samples for data normalization to overcome the large measurement batch effects between different datasets (9). Therefore, sample risk classification will be influenced by the risk composition of other samples adopted for data normalization. Additionally, the signature based on the quantitative expression measurements lacks robustness for quality uncertainties of clinical samples, including varied proportions of tumor epithelial cells in tumor tissues from different tumor locations in the body of the same patient (10), partial RNA degradation during specimen storage and preparation (11), and RNA amplification bias for minimum specimens (12).

In contrast, the within-sample relative expression orderings (REOs) of genes are the qualitative transcriptional characteristics of samples, which are robust against the experimental batch effects, and disease signatures based on REOs can be directly applied to individual level samples (8). Most importantly, we have reported that the within-sample REOs of genes are highly robust against the above-mentioned quality uncertainties of clinical samples, which are common factors that can lead to the failure of the quantitative transcriptional signature in clinical applications. Therefore, it is worthwhile to apply the within-sample REOs to find a robust qualitative transcriptional signature to predict the response states of platinum-taxane ACT for HGS-OvCa patients.

In this study, we combined the pathological response states and the recurrence-free survival (RFS) information to extract a qualitative transcriptional signature for predicting the RFS of patients receiving platinum-taxane ACT after surgery. The performance of the signature was validated in two independent datasets.

## Materials and Methods

### Data Sources and Data Pre-processing

In this study, one gene expression dataset of HGS-OvCa was downloaded from The Cancer Genome Atlas (TCGA, https://portal.gdc.cancer.gov/, 2017) and two gene expression datasets of HGS-OvCa were downloaded from the Gene Expression Omnibus (GEO, http://www.ncbi.nlm.nih.gov/geo/). TCGA expression profiles of 226 stage II-IV HGS-OvCa patients receiving platinum-taxane ACT after surgery was used to train a qualitative predictive signature. In the training dataset (TCGA), 163 patients were diagnosed as having a pathological complete response (CR), while the other 63 patients were diagnosed as having a pathological non-complete response (non-CR), including 32 partial responses, 17 stable disease, and 14 progressive disease patients (13). One independent dataset [GSE30161 (14)], denoted as test 1, recorded the RFS information and the pathological response states of patients receiving platinum-taxane ACT after surgery, including 22 CR patients and 16 non-CR patients (15 partial response and 1 progressive disease). The dataset was used to test the performance of the signature in predicting recurrence risk and pathological response state of patients receiving platinum-taxane ACT. Another independent dataset [GSE9891 (15)], denoted as test 2, which only provide the RFS information of 132 patients, was used to test the performance of the qualitative signature in predicting recurrence risk for patients receiving platinum-taxane ACT after surgery. Notably, all the primary tumor samples used in this study were extracted from the HGS-OvCa patients before receiving ACT. The clinical information of patients in the three datasets is described in Table 1.

**Table 1:** Clinical information of datasets analyzed in this study.

**Variable**		**Training**	**Test 1**	**Test 2**
Data source		TCGA	GSE30161	GSE9891
Sample size	Total	226	38	132
Treatment	Drug	Platinum-taxane	Platinum-taxane	Platinum-taxane
Survival data	–	RFS	RFS	RFS
Age	–	59 (34-87)	62 (44–78)	59 (23-79)
Grade	G2	30	14	56
	G3	196	24	76
Tumor stage	II	14	–	3
	III	181	34	118
	IV	31	4	11
Residual tumor	0–10 nm	109	14	76
	≥11 nm	55	23	43
	Un	62	1	13
Response state	CR	163	22	–
	Non-CR	63	16	–
	PR	32	15	–
	SD	17	0	–
	PD	14	1	-
Platform	–	Illu.HiSeqV2	Affy.U133 Plus2.0	Affy.U133 Plus2.0

*RFS, Recurrence-free survival; Un, unknown; CR, Complete response; Non-CR, Non-Complete response including Partial response (PR), Stable disease (SD), and Progressive disease (PD); Affy., Affymetrix; Illu., Illumina*.

For TCGA data derived from Illumina HiSeq 2000 RNA Sequencing Version 2 (Illumina, San Diego, CA, USA), the normalized count values determined by fragments per kilobase of exon per million fragments mapped (FPKM) method were obtained and log2-transformed was used for the gene expression. For data generated by Affymetrix platforms, the robust multi-array average algorithm (RMA) was used for pre-processing the raw data. Probe IDs were matched with Gene IDs using the corresponding platform files. For each sample, the expression measurements of all probe IDs corresponding to the same Gene ID were averaged to obtain a single measurement. Probes that did not match any Gene ID or that matched multiple Gene IDs were deleted (16).

### Survival Analyses

The RFS of patients were truncated at 5 years (60 months) such that patients with more than 5 years of follow-up were censored at 5 years. Survival curves were estimated using the Kaplan–Meier method and compared using the log-rank test. The univariate Cox proportional-hazards regression model was used to identify risk factors for recurrence of HGS-OvCa patients, including known prognostic clinical factors: age (≥60 vs. <60 years), stage (IV vs. III vs. II), histological grade (3 vs. 2) and residual tumor (≥11 vs. 0–10 mm). The multivariate Cox proportional-hazards regression model was used to evaluate the independent performance of the signature after adjusting for the clinical factors with *p*-value <0.2 in the univariate Cox model. Hazard ratios (HRs) and 95% confidence intervals (CIs) were generated using the Cox proportional hazards model. The concordance index (C-index) (17) was used to estimate the predictive performance of a signature for patient survival.

### Developing a Qualitative Predictive Signature for Platinum-Taxane ACT

Step 1: Finding prognosis-associated genes:

In the training data, Student's *t*-test with 5% *p* control was used to select the potentially differentially expressed genes (DE genes) between pathological CR and non-CR groups. From the potential DE genes, we selected the potential prognosis-associated genes whose gene expressions were significantly associated with patients' RFS using the univariate Cox model with 5% *p* control.

Step 2: Finding prognosis-associated gene pairs:

All possible gene pairs were constructed from both prognosis-associated genes. The samples could be classified into two groups according to the within sample REO (E*a* > E*b* or E*a* < E*b*) of each gene pair. Here, E*a* and E*b* represent the expression levels of two prognosis-associated genes, *a* and *b*, respectively. From all the possible gene pairs, we selected prognosis-associated gene pairs whose specific REO patterns (e.g., E*a* > E*b*) were significantly associated with longer RFS of patients using the univariate Cox model with 5% FDR control. For each prognosis-associated gene pair, the patients with the specific REO pattern (e.g., E*a* > E*b*) were voted as responders, otherwise (e.g., E*a* < E*b*) voted as non-responders. The C-index was used to evaluate predictive performance of each prognosis-associated gene pair.

Step 3: Developing a predictive gene pair signature:

Based on the prognosis-associated gene pairs, we applied a forward selection procedure to search for a signature that achieved the largest C-index value for predicting the RFS of patient receiving platinum-taxane ACT. Here, we chose each prognosis-associated gene pair as a seed and added to the optimal gene pair one at a time until the C-index did not increase. During the procedure, we adopted a simple majority-voting rule as follows: a sample was predicted as the responder, if more than half of the REOs (E*a* > E*b*) of gene pairs in the set voted as a responder; otherwise, it will be considered a non-responder. We selected the set of gene pairs that had the largest C-index in the procedure as a predictive gene pair signature (GPS) for platinum-taxane ACT.

### Statistics Analyses

The Chi-square test was used to examine the association of two response groups predicted by the GPS with the four known molecular subtypes of HGS-OvCa patients. Fisher's exact test was used to examine the association of two response groups predicted by the signature with the pathological response states. Student's *t*-test was used to examine the difference in levels of gene expression between the two response groups predicted by the signature. For exploring the biological function of DE genes, we conducted the gene functional enrichment analysis using the R package clusterProfiler (18), where a hypergeometric test, based on the current Kyoto Encyclopedia of Genes and Genomes (KEGG) databases, was employed. Significance was defined as *p* < 0.05 or FDR < 0.05 for multiple testing. All statistical analyses were performed using the R 3.4.3 (http://www.r-project.org/).

## Results

### Identification of a Predictive GPS for Platinum-Taxane ACT of HGS-OvCa Patients

**Figure 1:**
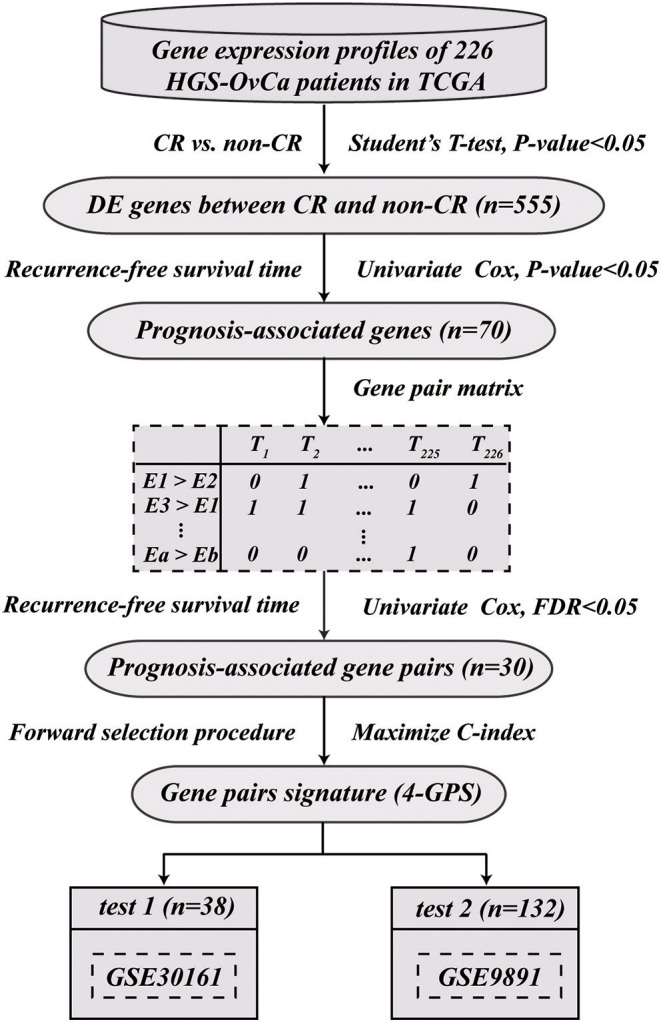
The flowchart for developing and validating the qualitative predictive GPS for platinum-taxane ACT.

**Figure 2:**
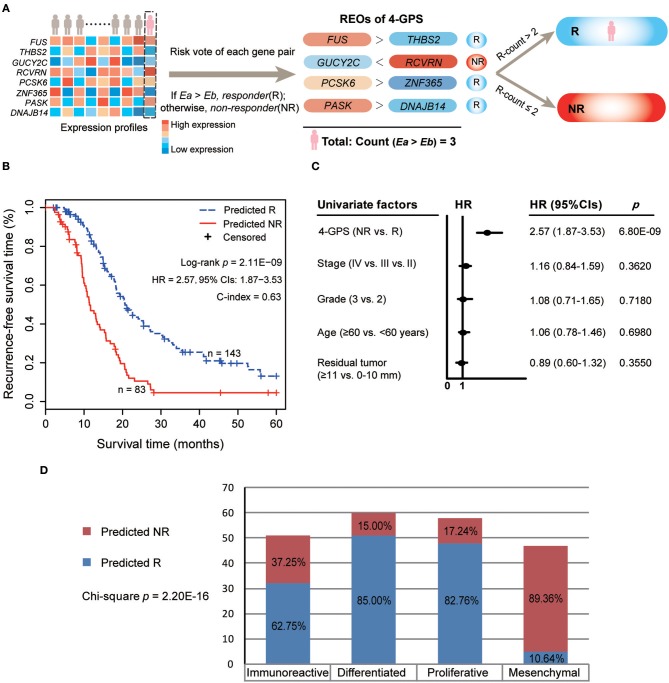
The predictive 4-GPS for risk classification and recurrence analyses for HGS-OvCa patients in TCGA. **(A)** Risk classification of 4-GPS for HGS-OvCa patients based on the within-sample relative expression orderings (REOs) of the four gene pairs with the majority-voting rule. A sample is predicted to be responder (R) if more than two of the four gene pairs have the specific REOs (E*a* > E*b*); otherwise, it will be predicted to be non-responder (NR). The sample exemplar (pink) is predicted to be responder because the count of the specific REOs (E*a* > E*b*) in the sample is 3. **(B)** The Kaplan–Meier curves of RFS for the 226 HGS-OvCa patients receiving platinum-taxane ACT in the training dataset. Hazard ratio (HR) and 95% confidence intervals (CIs) were determined using univariate Cox regression models. **(C)** Univariate Cox analyses of 4-GPS, age, stage, grade, and residual tumor. Solid circles represent the HRs for risk of recurrence, and the open-ended horizontal lines represent the 95% CIs. **(D)** The Confusion Matrix for the response prediction of 4-GPS and the HGS-OvCa molecular subtypes in TCGA. Chi-square test was used to compare the association.

**Figure 3:**
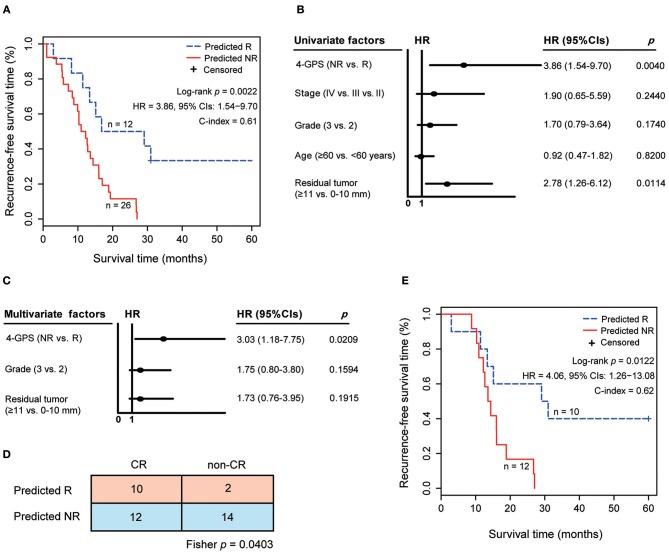
The validation of 4-GPS in test 1. **(A)** The Kaplan–Meier curves of RFS for 38 HGS-OvCa patients treated with platinum-taxane ACT. **(B)** Univariate Cox analyses of 4-GPS, age, stage, grade, and residual tumor in test 1. **(C)** Multivariate Cox analyses of 4-GPS after adjusting for significantly prognostic clinical factors in test 1. **(D)** The Confusion Matrix for the response prediction of 4-GPS with pathological response states. Fisher's exact test was used to compare the association. **(E)** The Kaplan–Meier curves of RFS for 22 HGS-OvCa patients receiving platinum-taxane ACT in the pathological complete response group.

Figure 1 describes the flowchart of this study. Here, we focused on analyzing the 9,819 genes commonly measured by the two platforms, IlluminaHiSeq_RNASeqV2 and Affymetrix U133 Plus 2.0, used in this study. First, we extracted 555 potential DE genes between 163 pathological CR and 63 pathological non-CR patients from the TCGA dataset (Student's *t*-test, *p* < 0.05). Among the potential DE genes, we pre-selected 70 potential prognosis-associated genes whose expressions were significantly associated with patients' RFS (univariate Cox model, *p* < 0.05). Next, from all the gene pairs constructed by the prognosis-associated genes, we identified 30 prognosis-associated gene pairs whose REOs were significantly associated with patients' RFS (univariate Cox model, FDR < 0.05). With a forward selection procedure using each of the 30 prognosis-associated gene pairs as a seed separately (see Materials and Methods), we obtained 30 sets of gene pairs, among which a set of four gene pairs reached the largest C-index of 0.63. Thus, the four gene pairs were selected as the predictive gene pair signature for platinum-taxane ACT, denoted as 4-GPS (Table 2). The classification rule of 4-GPS is that a sample was predicted as a responder, if more than two of the four gene pairs have the specific REOs (E*a* > E*b*); otherwise, it will be predicted to be a non-responder (Figure 2A). The R function for the classification of 4-GPS for a cohort or an individual is available in the Supplementary R function: HGS-OvCa response prediction for platinum-taxane ACT (see Supplementary Material). According to the majority voting rule, 83 patients were predicted as non-responders, and had significantly shorter RFS than 143 patients predicted as responders (log-rank *p* = 2.11E-09, HR = 2.57, 95% CIs: 1.87–3.53, C-index = 0.63, Figure 2B). A univariate Cox analysis showed that only 4-GPS (non-response vs. response, *p* = 6.80E-09, HR = 2.57, 95% CIs: 1.87–3.53, Figure 3C) was statistically significantly associated with patients' RFS. The univariate Cox result of the clinical factors are also displayed in Figure 3C.

**Table 2:** Composition and Biologic functional characterizations of 4-GPS.

**Signature REO (E*a* > E*b*)**	**HR**	***P*-value**	**C-index**	**Biologic functional characterizations**
Gene pair 1 (*FUS* > *THBS2*)	0.54	4.12E-4	0.57	*FUS*: A DNA/RNA-binding protein that plays a role in DNA repair and damage response (19).
				*THBS2*: This gene encodes a disulfide-linked homotrimeric glycoprotein that mediates cell-to-cell and cell-to-matrix interactions (20).
Gene pair 2 (*GUCY2C* > *RCVRN*)	0.47	5.61E-5	0.58	*GUCY2C*: This gene encodes a transmembrane protein that functions as a receptor for endogenous peptides guanylin and uroguanylin (21).
				*RCVRN*: This gene encodes a member of the recoverin family of neuronal calcium sensors. Recoverin may be the antigen responsible for cancer-associated (22).
Gene pair 3 (*PCSK6* > *ZNF365*)	0.57	7.96E-4	0.58	*PCSK6*: A pro-protein convertase that plays an important role in cancer cell proliferation (23).
				*ZNF365*: A DNA repair pathway gene in the homologous recombination pathway that is important in the repair of complex double-stranded lesions (24).
Gene pair 4 (*PASK* > *DNAJB14*)	0.55	2.66E-4	0.58	*PASK*: Alternatively spliced transcript variants encoding multiple isoforms have been observed for this gene (25).
				*DNAJB14*: Required to promote protein folding and trafficking, prevent aggregation of client proteins, and promote unfolded proteins to endoplasmic reticulum-associated degradation (ERAD) pathway.

*REO represents the relative expression ordering of gene pair (Ea > Eb); HR and P-value are the statistic calculated from the univariate Cox regression model. HR represents the risk coefficient of the REO for gene pair (a, b), where HR < 1 indicates that Ea > Eb is a protective factor, otherwise a risk factor; P-value represents the significance of the REO for gene pair (a, b)*.

Next, we analyzed the association of two response groups predicted by 4-GPS with the four molecular subtypes of the HGS-OvCa patients in the TCGA dataset, which were stratified by the TCGA original article (26). Here, 216 of 226 patients used in this study had molecular subtype information, which were classified as immunoreactive (*n* = 51), differentiated (*n* = 60), proliferative (*n* = 58), and mesenchymal (*n* = 47) subtypes (27). We found that 42 (89.36%) of 47 patients in the mesenchymal subtype were predicted to be non-responders by 4-GPS, with its proportion significantly higher than that in the other three subtypes (Chi-square test, *p* = 2.20E-16, Figure 2D). The result was in accordance with a previous report that shows that the mesenchymal subtype is associated with a poor prognosis of HGS-OvCa patients receiving platinum-taxane ACT after surgery (28), providing the biological evidence that 4-GPS has the ability to identify non-responders of platinum-taxane ACT.

### Independent Validation of 4-GPS

The performance of 4-GPS was tested in two independent datasets, which were detected in different laboratories with different microarray platforms.

In test 1, 26 non-responders predicted by 4-GPS had significantly shorter RFS than 12 predicted responders (log-rank *p* = 0.0022, HR = 3.86, 95% CIs: 1.54–9.70, C-index = 0.61, Figure 3A) after receiving platinum-taxane ACT. A univariate Cox analysis showed that 4-GPS (non-response vs. response, *p* = 0.0040, HR = 3.86, 95% CIs: 1.54–9.70, Figure 3B), histological grade (3 vs. 2, *p* = 0.1740, HR = 1.70, 95% CIs: 0.79–3.64, Figure 3B) and residual tumor (≥11 vs. 0–10 mm, *p* = 0.0114, HR = 2.78, 95% CIs: 1.26–6.12, Figure 3B) were significantly or marginally significantly associated with patients' RFS. Multivariate Cox analysis for 37 patients with complete clinical information showed that 4-GPS remained significantly associated with patients' RFS (*p* = 0.0209, HR = 3.03, 95% CIs: 1.18–7.75, Figure 3C), after adjusting for histological grade (*p* = 0.1594, HR = 1.75, 95% CIs: 0.80–3.80, Figure 3C) and residual tumor (*p* = 0.1915, HR = 1.73, 95% CIs: 0.76–3.95, Figure 3C). Notably, test 1 also provides patients' pathological response states for platinum-taxane ACT, including 22 pathological CR patients and 16 pathological non-CR patients. The result showed that the non-responders predicted by 4-GPS were significantly enriched in the pathological non-CR group (Fisher's exact test, *p* = 0.0403, Figure 3D). It is worth noting that 4-GPS reclassified 12 out of 22 pathological CR patients as non-responders and two out of 16 pathological non-CR patients as responders. In the pathological CR group, we found that 12 non-responders reclassified by 4-GPS had significantly shorter RFS than 10 responders consistently predicated by 4-GPS (log-rank *p* = 0.0122, HR = 4.06, 95% CIs: 1.26–13.08, C-index = 0.62, Figure 3E). The above result indicates a better classification of 4-GPS for platinum-taxane response states of HGS-OvCa patients. In addition, the accuracy of the two pathological non-CR patients reclassified as responders by 4-GPS needs further validation, as the small sample size is unfit for survival analysis.

**Figure 4:**
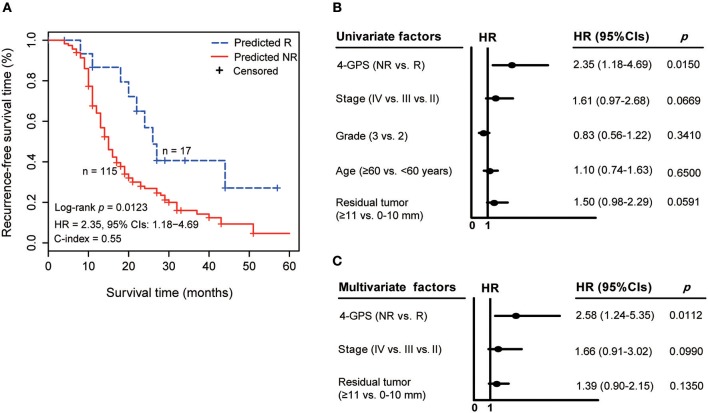
The validation of 4-GPS in test 2. **(A)** The Kaplan–Meier curves of RFS for 132 HGS-OvCa patients receiving platinum-taxane ACT in test 2. **(B)** Univariate Cox analyses of 4-GPS, age, stage, grade, and residual tumor in test 2. **(C)** Multivariate Cox analyses of 4-GPS after adjusting for significantly prognostic clinical factors in test 2.

Similarly, in test 2, 115 non-responders predicted by 4-GPS also had significantly shorter RFS than 17 predicted responders (log-rank *p* = 0.0123, HR = 2.35, 95% CIs: 1.18–4.69, C-index = 0.55, Figure 4A). A univariate Cox analysis showed that 4-GPS (non-response vs. response, *p* = 0.0150, HR = 2.35, 95% CIs: 1.18–4.69, Figure 4B), stage (IV vs. III vs. II, *p* = 0.0669, HR = 1.61, 95% CIs: 0.97–2.68, Figure 4B) and residual tumor (≥11 vs. 0–10 mm, *p* = 0.0591, HR = 1.50, 95% CIs: 0.98–2.29, Figure 4B) were significantly or marginally significantly associated with patients' RFS. And, a multivariate Cox analysis for 119 patients with complete clinical information also showed that 4-GPS remained significantly associated with patients' RFS (*p* = 0.0112, HR = 2.58, 95% CIs: 1.24–5.35, Figure 4C), after adjusting for the significant clinical factors including stage (*p* = 0.0990, HR = 1.66, 95% CIs: 0.91–3.02, Figure 4C) and residual tumor (*p* = 0.1350, HR = 1.39, 95% CIs: 0.90–2.15, Figure 4C).

### Functional Characterizations of 4-GPS

**Figure 5:**
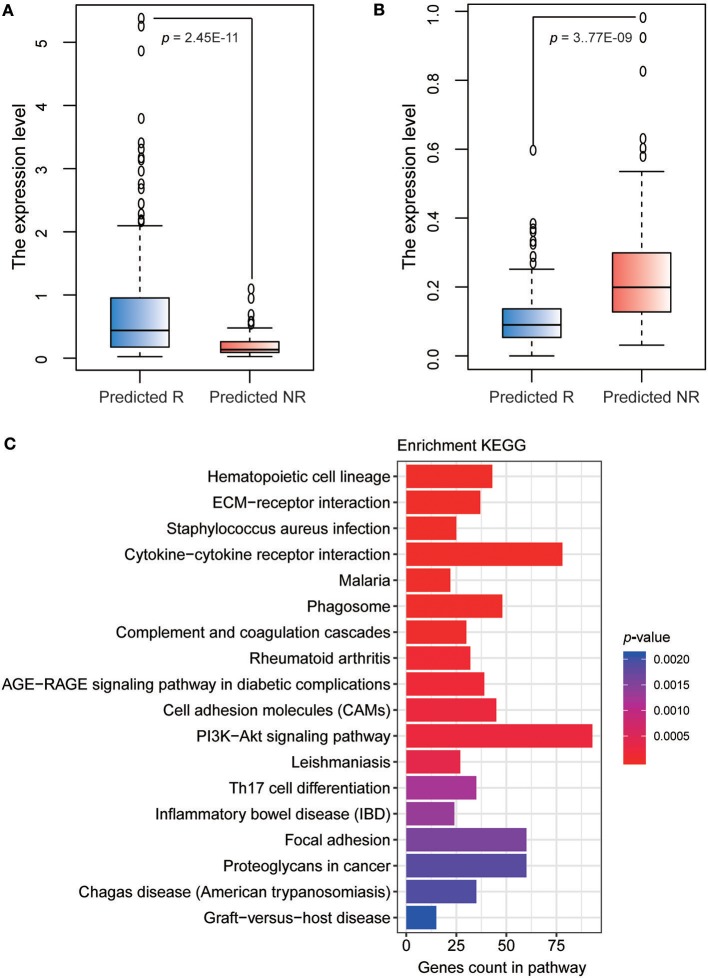
Functional characterizations of 4-GPS. **(A)** The boxplot of expression level of *PCSK6* in predicted response and non-response groups in the training dataset. **(B)** The boxplot of expression level of *ZNF365* in predicted response and non-response groups in the training dataset. **(C)** KEGG functional enrichment analyses of DE genes between non-responders and responders predicted by 4-GPS in the training dataset.

The detailed information of 4-GPS is described in Table 2. Functional annotation showed that six signature genes involved in all four gene pairs were included in cell adhesion and regulation of angiogenesis [*THBS2* (20)], cell proliferation [*GUCY2C* (21)], and the regulation of cellular and metabolic processes [*FUS* (19); *PASK* (25); *PCSK6* (29); *RCVRN* (22)], which have been reported to be related with platinum and/or taxane sensitivity. Several genes including *ZNF365* (30), *THBS2* (20), *GUCY2C* (21), *PCSK6* (29), and *RCVRN* (22), have been reported to be associated with a poor prognosis of HGS-OvCa patients or other cancer patients treated with ACT after surgery. The REO of two genes in a gene pair has intuitive biological implications in tumor progression. For example, *PCSK6*, a pro-protein convertase, plays an important role in cancer cell proliferation (23) and *ZNF365*, a DNA repair pathway gene in the homologous recombination (HR) pathway (24). In the training dataset, the expression level of *PCSK6* in the predicted response group was significantly higher than that in the predicted non-response group (Student's *t*-test, *p* = 2.45E-11, Figure 5A), while the expression level of *ZNF365* in the predicted response group was significantly lower than that in predicted non-response group (Student's *t*-test, *p* = 3.77E-09, Figure 5B). Therefore, the relative order of *PCSK6* expression level was higher than that of *ZNF365* in the predicted responders and reversed in the predicted non-responders. This indicates that the responders predicted by 4-GPS might have higher cell proliferative capacity and lower DNA repair capacity than the predicted non-responders. Therefore, the cancer cells in the predicted responders were more easily attacked by platinum agents and could not repair the lesions induced by platinum agents.

The functional enrichment analyses also supported the ability of 4-GPS in distinguishing the response and non-response to platinum-taxane ACT. In the training dataset, we identified 2,410 DE genes between 83 non-responders and 143 responders predicted by 4-GPS (Student's *t*-test, FDR < 0.05). The DE genes were significantly enriched in 18 KEGG functional terms (hypergeometric test, FDR < 0.05, Figure 5C), including several functions related with platinum resistance for HGS-OvCa patients, such as PI3K-Akt signaling pathway (31), and cell adhesion molecules (32). In addition, some other functions have been reported to be related to taxane sensitivity for HGS-OvCa patients, such as focal adhesion (33), and cytokine-cytokine receptor interaction (34).

### Comparison of 4-GPS With Other Signatures

**Figure 6:**
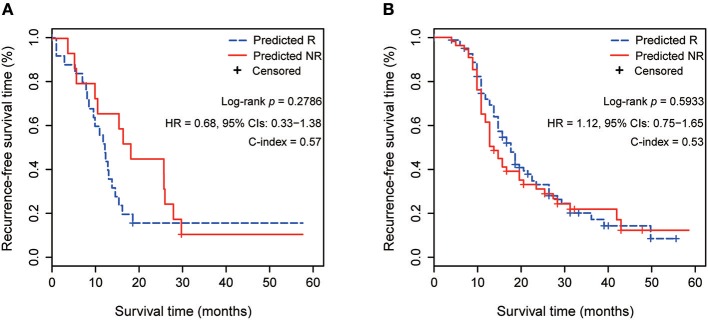
Prognostic performance of 23-gene signature in 2 test datasets. **(A)** The Kaplan–Meier curves of RFS for 38 HGS-OvCa patients treating with platinum-taxane ACT in test 1. **(B)** The Kaplan–Meier curves of RFS for 132 HGS-OvCa patients treating with platinum-taxane ACT in test 2.

We also compared the performance of 4-GPS with the published 23-gene signature in test 1 and test 2 of this study, respectively, which were not the training datasets for the two signatures. The 40-gene signature (6) cited in the introduction was not analyzed because its application to independent data needs resetting risk thresholds, which makes it a non-independent validation. The other published signatures, such as the 422-gene signature (4), were not analyzed because the author did not provide the predictive model. Briefly, for 23-gene signature (5), a point of each sample was given for each gene if its high expression was associated with longer (or shorter) survival in its training dataset and if its expression in the sample was higher (or lower) than the median expression of all samples. The risk score was the sum of these points calculated by 23 genes. The samples were categorized as a high-risk (non-response) group when their scores were lower than 11 (training cut-off), and vice versa. The survival results showed that 23-gene signatures failed to predict the RFS of patients receiving platinum-taxane ACT in the two test datasets (Figures 6A,B). Moreover, 23-gene signature could not predict the response states of individual samples when no other samples were analyzed together for comparison. The requirement of a comparison with the other samples needs pre-collection of a set of samples, and the risk prediction of an individual sample will rely on the risk composition of other samples adopted for comparison. This provided further evidence that the type of quantitative signatures would be unfit to direct clinical settings, as reported in our previous study (8).

## Discussion

In this study, we constructed a qualitative transcriptional signature consisting of four gene pairs (4-GPS) by combining the pathological response state and RFS information of HGS-OvCa patients. The signature could identify the individual platinum-taxane responders with longer RFS after receiving platinum-taxane ACT, and its performance was effectively validated in two independent datasets.

According to response evaluation criteria in solid tumors (RECIST), a certain percentage of pathological response states of HGS-OvCA patients may be misclassified by the conventional iconographies, especially near the cut-off points for the short-term reduction of tumor size after platinum-taxane ACT (35). Therefore, the National Comprehensive Cancer Network (NCCN) clinical guidelines for HGS-OvCa patients recommend that patients with a pathological complete response but relapse within 6 months after receiving platinum-taxane ACT should be deemed resistant. In this study, we found that 4-GPS could reclassify 12 clinical diagnosed CR patients as non-responders in test 1 and their RFS was significantly shorter than the other CR patients (log-rank *p* = 0.0122; Figure 3E), suggesting a better performance of 4-GPS in identifying patients who are resistant to platinum-taxane.

The standard treatment for HGS-OvCa is surgery and platinum-based ACT, which is typically administered concurrently with a taxane (2). Recently, our study has proved that genes related to single drug sensitivity could be identified in clinical samples of patients who received a combination of ACT. This is because the drugs used in combination had no or limited pharmacological antagonism (36). In this study, we developed a predicted signature based on the patients receiving platinum combined with taxane. We considered that the responders predicted by the signature could be sensitive to either platinum or taxane, and should receive platinum combined with taxane ACT. While, the predicted non-responders could be resistant to both platinum and taxane, require further testing (such as BRCA mutation), and receive alternate therapies [e.g., bevacizumab (37)] after surgery.

**Figure 7:**
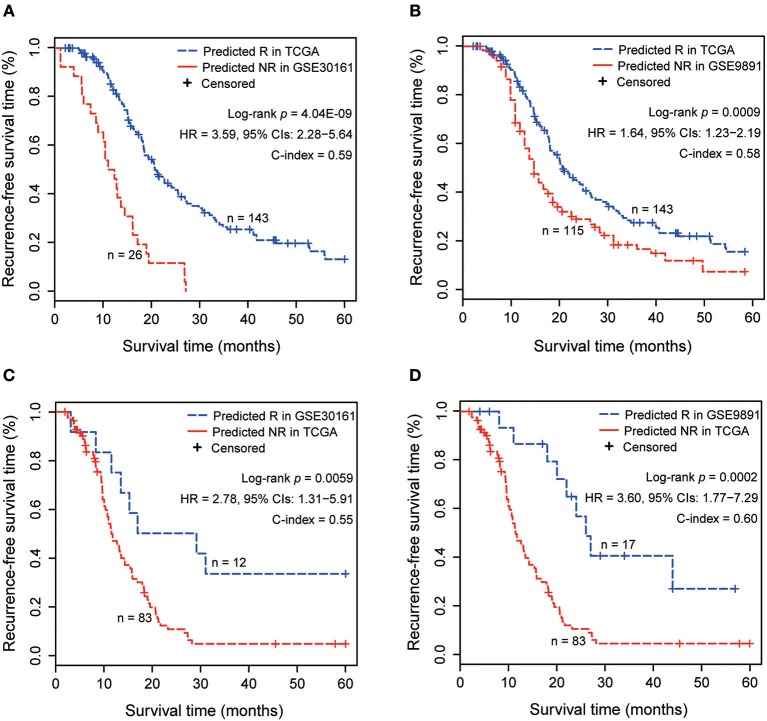
The cross comparisons of RFS between the predicted responders and non-responders derived from different cohorts. **(A)** The Kaplan–Meier curves of RFS for 143 responders predicted by 4-GPS in the training dataset and 26 non-responders predicted in test 1. **(B)** The Kaplan–Meier curves of RFS for 143 responders predicted by 4-GPS in training dataset and 115 non-responders predicted in test 2. **(C)** The Kaplan–Meier curves of RFS for 83 non-responders predicted by 4-GPS in the training dataset and 12 responders predicted in test 1. **(D)** The Kaplan–Meier curves of RFS for 83 non-responders predicted by 4-GPS in the training dataset and 17 responders predicted in test 2.

Notably, there were large differences in the predicted response/non-response ratios among cohorts. A previous study reported that different cohorts collected in the datasets had different risk compositions (8), such as the ratios of patients with potential metastases or resistance to drugs in the cohort. In order to support the accuracy of the signature in each dataset, we additionally performed the cross comparison of RFS between the predicted responders and non-responders derived from different cohorts. The results showed that the responders predicted by 4-GPS in the training data had significantly longer RFS than the non-responders predicted in test 1 (log-rank *p* = 4.04E−09, Figure 7A) and test 2 (log-rank *p* = 0.0009, Figure 7B), and the predicted non-responders also had significantly shorter RFS than the responders predicted in test 1 (log-rank *p* = 0.0059, Figure 7C) and test 2 (log-rank *p* = 0.0002, Figure 7D). Similar results for the cross comparisons were observed in test 1 and test 2 (Figure S1). The results also provided the indirect evidence for the rationality of the large difference in predicted responders/non-responders among different cohorts.

In conclusion, the qualitative predictive signature could be applied to the gene expression profile of the postoperative sample, obtained from an individual, to determine the response state for the platinum-taxane ACT. For the predicted responders, they should be advised to receive platinum combined with taxane ACT after surgery, while for the predicted non-responders, they should be evaluated for the other therapies, which requires for further exploration. The signature is highly robust against experimental batch effects and uncertainties of quality of clinical samples, which is convenient in clinical settings and requires further validation in a prospective clinical trial.

## Data Availability Statement

Publicly available datasets were analyzed in this study. This data can be found here: https://portal.gdc.cancer.gov/, https://www.ncbi.nlm.nih.gov/geo/query/acc.cgi?acc=GSE30161, https://www.ncbi.nlm.nih.gov/geo/query/acc.cgi?acc=GSE9891.

## Author Contributions

ZG and LQ conceived the idea. LQ and YLiu conceived and designed the experiments and wrote the manuscript. YLiu and ZZ designed the experiments. YLi, ZZ, TL, XL, and SZ performed the experiments and analyzed the data. YG and WZ helped in interpreting the results and writing the manuscript. All authors approved the final version.

### Conflict of Interest

The authors declare that the research was conducted in the absence of any commercial or financial relationships that could be construed as a potential conflict of interest.
