# Supplementary material for: A Qualitative Transcriptional Signature for Predicting Recurrence Risk for High-Grade Serous Ovarian Cancer Patients Treated With Platinum-Taxane Adjuvant Chemotherapy
Source: Front Oncol. 2019 Oct 18;9:1094. doi: 10.3389/fonc.2019.01094 (PMC6813654; doi:10.3389/fonc.2019.01094)
Supplement: Supplementary file 1 [file Data_Sheet_1.pdf]

Supplementary Material

1 Supplementary Figure S1

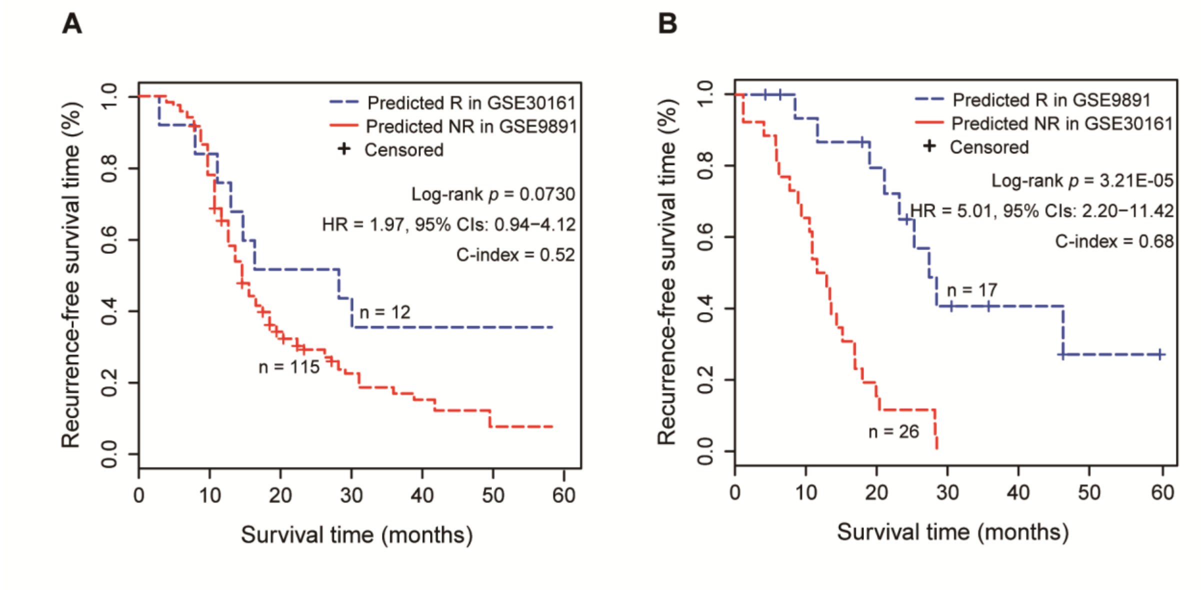

Supplementary Figure S1. The cross comparisons of RFS between the predicted responders and non-responders derived from test 1 and test 2 datasets. (A) The Kaplan–Meier curves of RFS for the 12 responders predicted by 4-GPS in test 1 and the 115 non-responders predicted in test 2. (B) The Kaplan–Meier curves of RFS for the 26 non-responders predicted in test 1 by 4-GPS and the 17 responders predicted by in test 2.

2 Supplementary R function: HGS-OvCa response prediction for platinum-taxane ACT.

R Codes for predicting recurrence risk for high-grade serous ovarian cancer patients treated with platinum-taxane adjuvant chemotherapy

(HGS-OvCa response prediction for platinum-taxane ACT)

Sep 16, 2019

```
# Description

#Response prediction of high-grade serous ovarian cancer (HGS-OvCa) for platinum-taxane adjuvant chemotherapy (ACT) by 4-GPS. The classification rule of 4-GPS is that a sample was predicted as responder, if more than two of the four gene pairs vote for response; otherwise, it will be predicted to be non-responder.

#

# Input

# exp: A matrix of expression profiles. The whole gene expression profile or the signature's gene expressions of a cohort or an individual

# geneList: A vector of gene list

# KeyType: Input gene type which must be one of "ENTREZID", "ENSEMBL" or "SYMBOL"

#

# Output

# The predicted recurrence risk for HGS-OvCa patients treated with platinum-taxane adjuvant chemotherapy

#

# Examples

# source('/path/RecurrenceRiskPredict.R')

# RESULTriskPredict <- 'RecurrenceRiskPredict (exp, geneid, 'ENTREZID')

######################################################################################

'RecurrenceRiskPredict <- function(exp, geneList, KeyType) {

  if (KeyType == 'ENTREZID') {

    GPS4 <- data.frame(Gene1=c(2521, 2984, 5046, 23178),
```

```
                        Gene2=c(7058, 5957, 22891, 79982),
                        stringsAsFactors = F)
  }else if (KeyType == 'ENSEMBL') {
    GPS4 <- data.frame(Gene1=c('ENSG00000089280', 'ENSG00000070019', 'ENSG00000140479', 'ENSG00000115687'),
                        Gene2=c('ENSG00000186340', 'ENSG00000109047', 'ENSG00000138311', 'ENSG00000164031'),
                        stringsAsFactors = F)
  }else if (KeyType == 'SYMBOL') {
    GPS4 <- data.frame(Gene1=c('FUS', 'GUC2C', 'PCSK6', 'PASK'),
                        Gene2=c('THBS2', 'RCVRN', 'ZNF365', 'DNAJB14'),
                        stringsAsFactors = F)
  }
  index1 <- match(GPS4$Gene1,geneList)
  index2 <- match(GPS4$Gene2,geneList)
  reo <- as.matrix(sign(exp[index1,] - exp[index2,]))
  score <- apply(reo, 2, function(x){sum(x==1)})
  label <- rep('Non-response',ncol(exp))
  label[score>2] <- 'Response'
  out <- data.frame(RiskScore=score, RiskCategory=label)
  return(out)
}
```
